# A vibrating beam MEMS accelerometer for gravity and seismic measurements

**DOI:** 10.1038/s41598-020-67046-x

**Published:** 2020-06-26

**Authors:** Arif Mustafazade, Milind Pandit, Chun Zhao, Guillermo Sobreviela, Zhijun Du, Philipp Steinmann, Xudong Zou, Roger T. Howe, Ashwin A. Seshia

**Affiliations:** 1 Nanoscience Centre, Department of Engineering, University of Cambridge, Cambridge, CB3 0FF UK; 2 Silicon Microgravity Ltd., Cambridge Innovation Park, Waterbeach, Cambridge, CB25 9GL UK; 3 State Key Lab of Transducer Technology, Institute of Electronics, Chinese Academy of Sciences, Beijing, 100190 China; 4 Department of Electrical Engineering, Stanford University, Stanford, CA 94305 USA

**Keywords:** Electrical and electronic engineering, Mechanical engineering, Applied physics, Geophysics

## Abstract

This paper introduces a differential vibrating beam MEMS accelerometer demonstrating excellent long-term stability for applications in gravimetry and seismology. The MEMS gravimeter module demonstrates an output Allan deviation of 9 μGal for a 1000 s integration time, a noise floor of 100 μGal/√Hz, and measurement over the full ±1 g dynamic range (1 g = 9.81 ms^−2^). The sensitivity of the device is demonstrated through the tracking of Earth tides and recording of ground motion corresponding to a number of teleseismic events over several months. These results demonstrate that vibrating beam MEMS accelerometers can be employed for measurements requiring high levels of stability and resolution with wider implications for precision measurement employing other resonant-output MEMS devices such as gyroscopes and magnetometers.

## Introduction

Recent advances in microelectromechanical systems (MEMS) have enabled the widespread development of sensors for a variety of consumer, automotive, and wearable healthcare electronics applications. Highly precise instruments, *e.g*., the atomic force microscope^1^ and ultra-stable clocks^2^ have also employed advances in MEMS technology for precision manufacturing of sensitive transducers and chip-scale system integration. MEMS technology combines miniaturization benefits (size, weight, and power) with a scalable manufacturing platform. Inertial sensors, in particular, have seen widespread application in the consumer and automotive electronics industries; there is increasing interest in developing highly precise accelerometers for seismic^3^ and gravity measurements^4,5^, as well as for navigation systems for semi-autonomous vehicles and pedestrians. Further applications for portable high-precision MEMS gravimeters and seismometers include monitoring of natural hazards, geotechnical surveying, and space missions. In this context, Lewis *et al*. recently demonstrated a remote surface gravity traverse on Mars using compact and robust instrumentation based on MEMS accelerometers integrated as part of the inertial measurement unit (IMU) on the Curiosity rover^6^; surveys on future planetary missions will require sensors with significantly higher accuracies, as well as the potential to combine background seismic measurements within the same module in alignment with device specifications reported in this paper.

Conventional MEMS accelerometers are typically based on a mass-spring system^3–5^, where design considerations concerning sensitivity and signal-to-noise run counter to dimensional scaling. A simple analysis based on Hooke’s law (see supplementary text) shows that the sensitivity of traditional accelerometers based on displacement-measurement scales inversely as the mass of the device or the inverse squared of the natural frequency^4^, which is counter to device robustness^7^ and miniaturization. Additionally, nonlinear springs^4,5^ are often employed in the engineering of such devices (in order to reduce the natural frequencies to a few Hz) introducing a significant additional calibration burden and an intrinsically limited measurement dynamic range. Furthermore, the potential to combine sensitive measurements over a wide dynamic range (> ±1 g) is a desirable feature in differential gravimeters in order to address inherent sensitivity to tilt and compensate for Earth’s background gravity field in terrestrial measurements.

Macroscopic accelerometers based on vibrating elements^8,9^ have previously been employed for applications demanding both accuracy and robustness. The early devices consist of a suspended mass connected to a vibrating wire under tension, such that the inertial force experienced by the mass due to gravity or an external acceleration is communicated as a variation in the axial tension of the wire, thereby modulating its natural frequency of vibration. The natural frequency of the vibrating element is continuously tracked through the implementation of a feedback oscillator circuit whose output frequency is proportional to the magnitude of the applied acceleration. Gilbert *et al*. proposed a vibrating wire gravimeter in 1949 for borehole measurements based on this principle^9^. Vibrating wire accelerometers were further developed for ship-borne^10^ and lunar gravimetry^11^.

More recently, vibrating beam resonant accelerometers have been miniaturised using silicon MEMS technology^12–14^ operating on similar principles. These MEMS accelerometers and other previous variants based on resonant sensing principles demonstrated significant drift from uncompensated environmental effects, particularly temperature variations, making them unsuitable for long-term, precision measurements. In this paper, we describe the development of the first MEMS vibrating beam accelerometer (VBA) that meets the stability requirements for use as a relative gravimeter and long-period seismometer. The sensing principle is based on monitoring resonant frequency shifts in vibrating beams, with drift compensated using a differential frequency readout configuration and active, chip- and module-level temperature control. The devices, which are fabricated in a silicon MEMS foundry using standard processes, demonstrate excellent stability over long integration times and are robust enough for field use. As opposed to mass-spring accelerometers, the scale factor of a vibrating beam gravimeter is length scale-invariant to first order (see Supplementary Information) and therefore, miniaturization does not have a detrimental impact on sensitivity or robustness.

### Device fabrication and assembly

**Figure 1:**
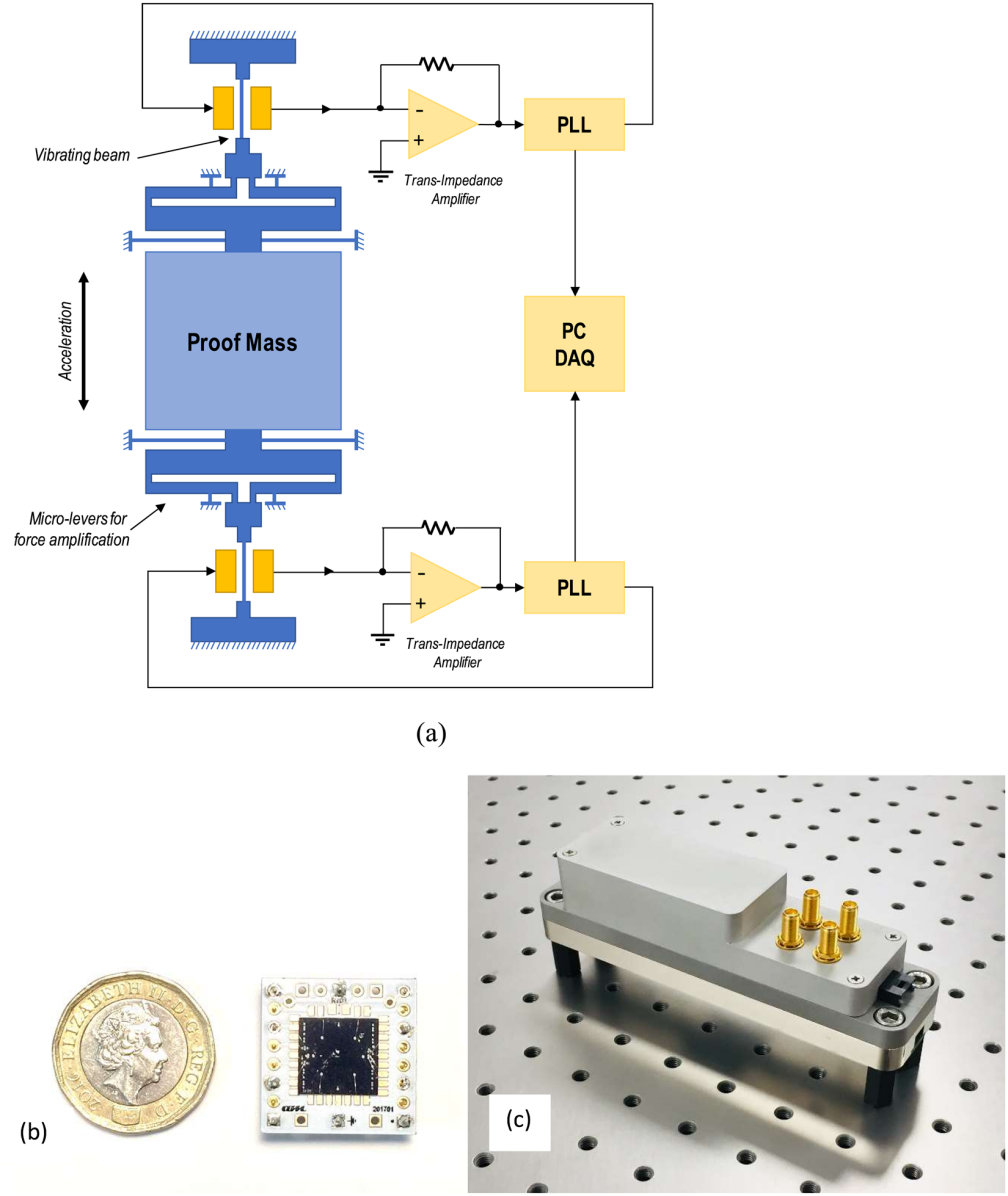
Schematic of accelerometer (**a**) and images of the packaged chip (**b**), and integrated sensor module (**c**).

The mechanical element of the sensor consists of a spring-supported mass with a natural frequency of approximately 1 kHz (Fig. S2) micromachined from the silicon device layer of a silicon-on-insulator (SOI) wafer^15^ using a deep-reactive ion etch process^16^. The sensor mass is approximately 6.83 milli-grams and the spring constant for translational motion along the sensitive axis is approximately 260 N/m. This mass is mechanically linked to the vibrating beam elements through a lever mechanism^14^ such that the axial force on the beams is proportional to the inertial force on the mass amplified by a factor of approximately 25. The vibrating beams are driven by electrodes located on either side of the beam, as illustrated schematically in Fig. 1(a). These beams can be driven in either the fundamental or the second-order flexural mode of vibration (see Fig. S3) using electrostatic actuation; the higher-order mode enables a further increase in scale factor for a given topology. Two nominally identical beams are located on either side of the mass, to provide a differential push-pull output response. Integrated parallel-plate capacitive transducers enable recording of the motional response of the vibrating beams.

Fusion bonding of silicon wafers^16^ is employed to seal the device within a vacuum environment, with the device layer sandwiched within a three-wafer stack (Fig. S1) and sealed in a stable, low-pressure vacuum environment^17^. Through-silicon-via (TSV) electrical interconnects^18^ connect structures on the device wafer to external electronics and a top wafer is bonded to achieve vacuum encapsulation of the suspended proof mass and attached resonators. The wafer-level vacuum encapsulation provides a stable local environment for the devices and reduces the impact of fluid drag^19^ so that the quality factor *Q* of the vibrating beam is typically higher than 30,000 for both the fundamental and second order flexural modes. The *Q* of the beams is primarily limited by a combination of thermo-elastic damping^20^ and energy loss through the anchors.

Following device fabrication, the wafer is diced and individual die are assembled onto custom chip carriers, as pictured in Fig. 1(b). The chip carrier consists of a heater element located underneath for temperature control. A copper mounting block is integrated on the top surface to provide shielding. The carrier is assembled onto a PCB stack and interfaced to custom front-end electronics, which consists of low-noise transimpedance amplifiers and a digital phase-locked loop to continuously track shifts in resonant frequency. Two output channels (one corresponding to each detector beam) are simultaneously recorded to provide continuous tracking of the resonant frequencies. A schematic of the device and associated front-end electronics is shown in Fig. 1(a). Temperature control of the sensitive external analogue front-end electronics is implemented within the sensor module (Fig. 1(c)) while local thermal control of the accelerometer chip^21^ enables active cancellation of the temperature-induced drift of the natural frequency. The drift arises due to the temperature sensitivity of the material properties of single-crystal silicon and can be reduced through additional active and passive compensation schemes^22^. A dedicated PC running National Instruments LabVIEW acquires the data generated from the module and consists of a custom frequency counter implemented to record the frequencies of the two channels to an accuracy of better than 1 μHz (Fig. S10). The data acquisition interface also allows simultaneous recording of temperature (at various locations within the sensor module, including the chip temperature), pressure, and power consumption of the temperature controllers to enable further corrections to be applied to the data through post-processing.

## Results

The devices are initially characterised on a tilt platform^14^, in which the sensitive axis is oriented at specified tilt angles relative to the vertical to obtain an estimate of the scale factor and also to characterise the tilt sensitivity of the device. The tilt sensitivity arises due to the large background gravitational field and varies as the cosine of the angle between the sensitive axis of the device and true local vertical as defined by the direction of the background field^23^. Next the output from the device is recorded with the sensitive axis aligned to the horizontal position without the external temperature control operating. The noise spectrum for this measurement is provided in Fig. S10 demonstrating a noise floor of 25 nano-g/√Hz at 1.5 Hz. Finally, the device is oriented with the sensitive axis aligned to the vertical^4^ with the external temperature control now operational to obtain data demonstrating sensitivity to tidal effects. The testing was conducted in a Cambridge University lab with significant background seismic noise, a lower noise basement laboratory at the University of Bristol, as well as at a low-noise observatory in Eskdalemuir, Scotland.

**Figure 2:**
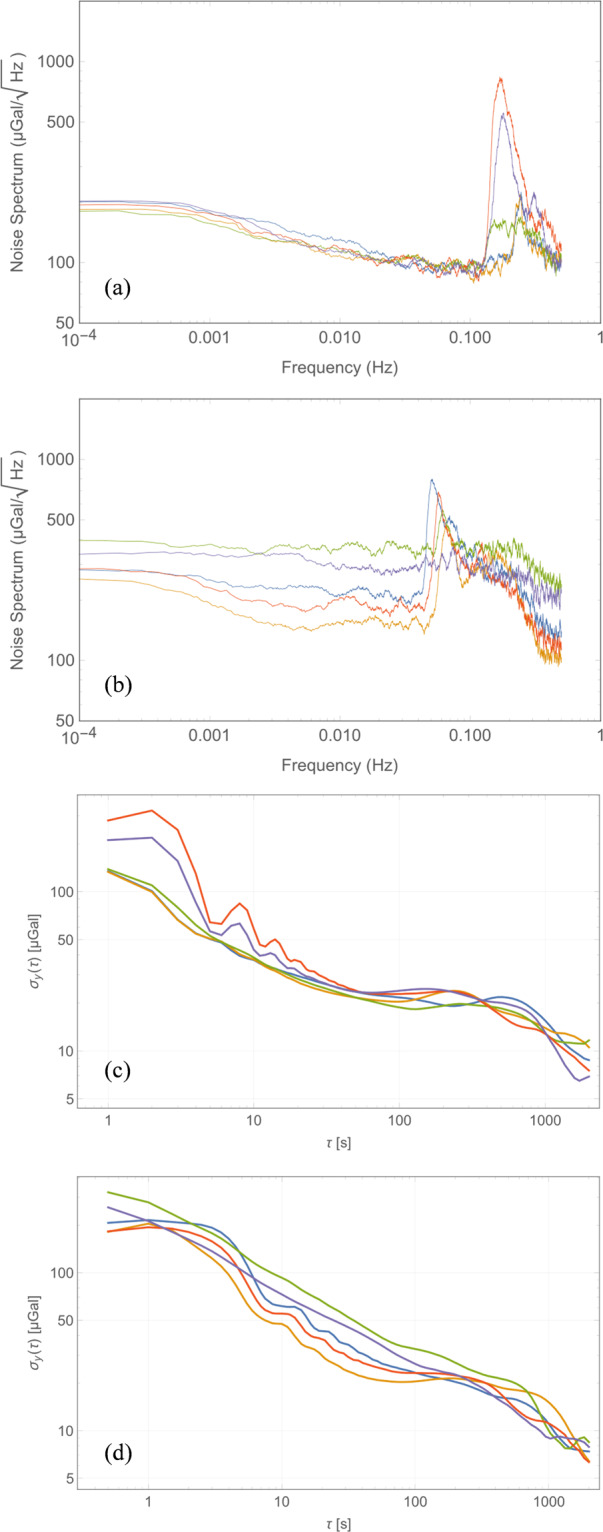
Accelerometer noise power spectral density measurement (five independent repeat measurements overlaid) for responses recorded (**a**) in a seismic vault at Eskdalemuir, Scotland demonstrating a noise floor of <100 μGal/rt-Hz, and (**b**) in the Cambridge University lab. The corresponding Allan deviation data has been plotted in (**c**),**d**) demonstrating a bias stability of <10 μGal for an integration time >1000 s following linear drift correction.

**Figure 3:**
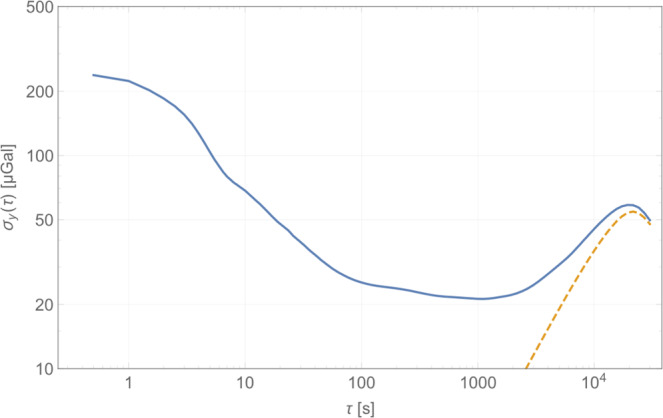
Allan deviation data showing tracking of Earth tides in the seismic vault at Eskdalemuir, Scotland. The statistical correlation coefficient R between the measured time series and the predicted Earth tide model (TSoft)^27^ is 0.862 over this period for this dataset. The blue solid lines show the Allan deviation for the MEMS sensor with the dashed orange lines plotting the Allan deviation for the theoretical data generated by TSoft.

**Figure 4:**
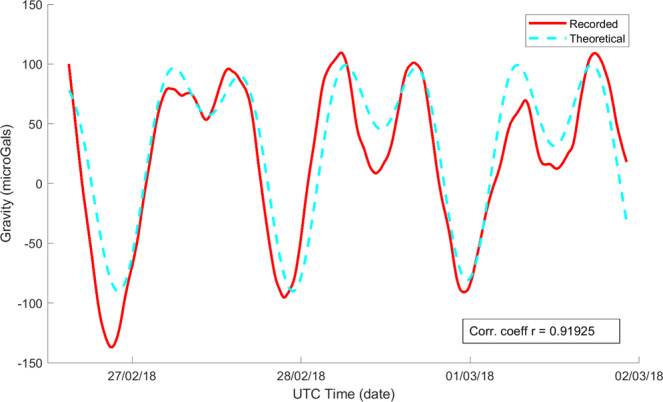
Data showing tracking of Earth tides over a four day period at the University laboratory. The solid line indicates the conditioned MEMS dataset and the dashed line indicates the theoretical prediction from TSoft with an ocean loading correction applied. The statistical correlation coefficient R between the measured time series and the predicted Earth tide model (TSoft)^27^ is 0.92 over this period.

**Figure 5:**
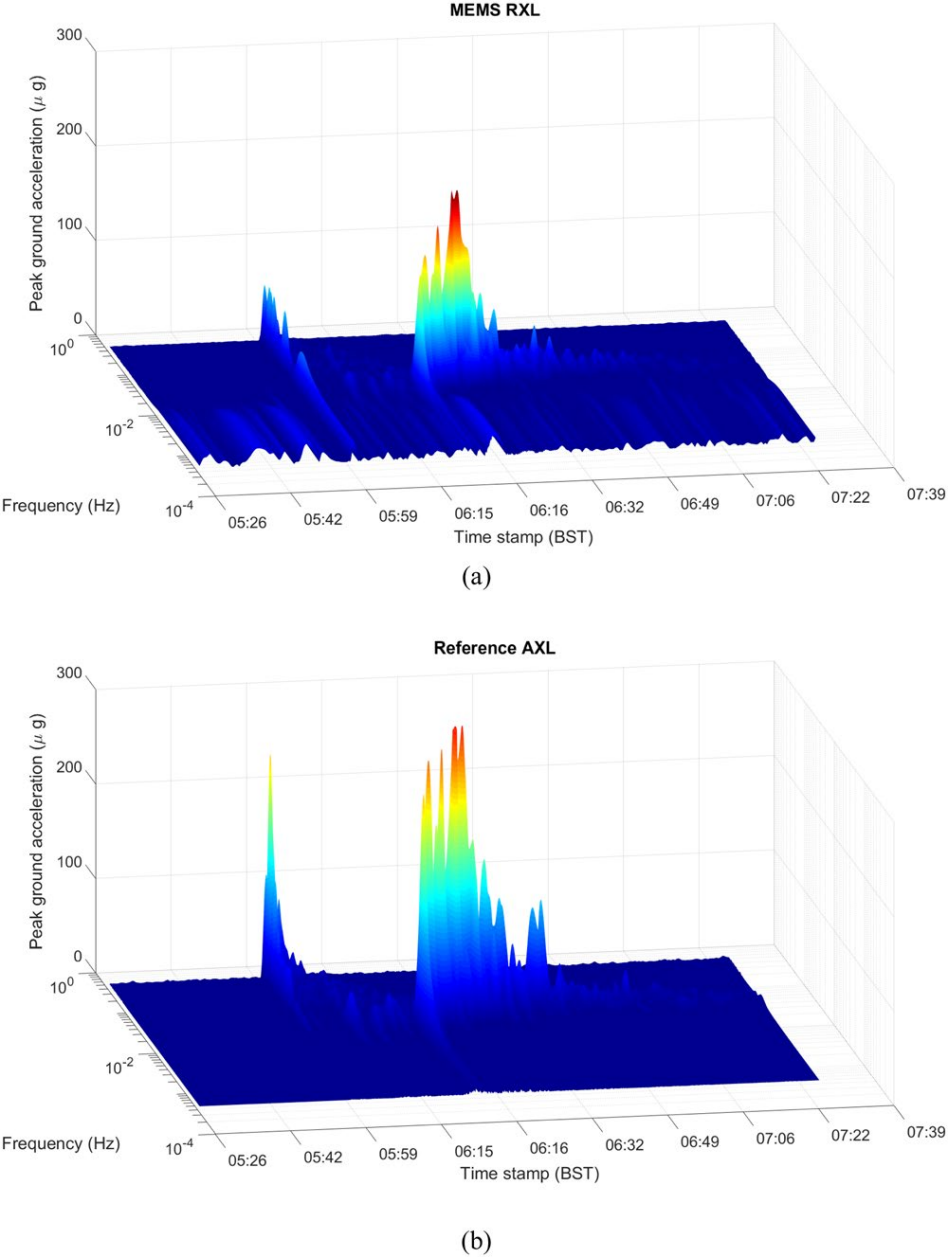
Spectrograms of the measured ground acceleration for (**a**) the MEMS sensor module located in a basement laboratory in Bristol, UK, and (**b**) a reference seismometer located in Swindon, UK, for the 2017 Chiapas, Mexico, Earthquake (Mw 8.2) on September 8 2017. The MEMS accelerometer output was sampled at 1 Hz while the reference accelerometer was sampled at 50 Hz.

The experimentally measured sensor scale factor for the selected device is 5972 Hz/g when the beams are operated in the second mode, close to the simulated value (Fig. S4). Figure 2(a) plots the acceleration noise power spectral density (PSD) of the device measured at Eskdalemuir, while Fig. 2(b) plots this for measurements from the Cambridge University lab with 5 independent datasets in each location overlaid on the same plot to demonstrating the repeatability of the measurements at different times under ambient test conditions. The noise floor of the fully integrated module is seen to be below 100 μGal/√Hz when the device is mounted vertically and measured in a low-noise seismic vault, with a higher noise floor recorded in the noisier University lab environment, due to the ambient seismic background. When operated continuously, a burn-in period of approximately two weeks was required prior to the device response exhibiting a continuous linear drift of approximately ~4 mHz/hour. This drift is cancelled in software by fitting to the linear trend and recovering the residual variations. The linear drift is thought to arise due to a variety of aging effects, not intrinsic to the device but due to package-induced stress, which has been shown to impact the drift characteristics for MEMS resonators^24^. Once the linear drift is cancelled, which is standard practice for relative gravimeters^4,5^, it is seen that the response is extremely stable, enabling gravity and seismic measurements over long time scales (100 s – 200000 s). Figure 2(c,d) plot the Allan Deviation^25^ of the output response over a 10000 s period for the same datasets (the Allan Deviation of the response prior to drift correction is plotted in Fig. S6), showing that the high frequency noise is averaged out over a period of 1000 s representative of the recording period for a single surface gravity measurement. An output Allan deviation of 9 μGal at 1000 s is recorded for one of the Cambridge University lab datasets. A similar stability is recorded when the device is run in a seismic vault operated by the British Geological Survey (Fig. 2(d)) with the micro-seismic background^26^ having prominent peaks in the frequency range 0.1 Hz – 0.5 Hz, which are also seen in the acceleration noise power spectrum plots. The device was then left running for a period of several days at the University lab and about 36 hours at the seismic vault for longer duration measurements. The output response from these measurements demonstrates tracking of the variations in the gravitational field due to Earth tides. Figure 3 plots the Allan deviation of the response measured in the seismic vault over the 36 hour period with the drift-compensated data averaged over a period of 12800 s, similar to the timescale reported in^4^. This is compared to the data obtained from the software TSoft^27^ and a statistical correlation R of 0.86 is noted for this dataset. Longer duration measurements were possible in the University lab where a statistical correlation R of 0.92 is noted between the conditioned MEMS dataset (see Methods section) and the predicted Earth tides model from TSoft following correction for ocean loading effects over a period of 4 days as shown in Fig. 4. Longer timescale measurements over the course of 6 days demonstrated a lower statistical correlation R of 0.88 due to the residual nonlinearity in the drift over this duration. The device also recorded the ground motion from a number of teleseismic events during the recording period, prominent among them being the Chiapas, Mexico earthquake on September 7, 2017 at the University of Bristol basement laboratory, demonstrating excellent performance as a long-period seismometer. Spectrograms corresponding to the recorded response of the MEMS sensor for this Mw8.2 event are compared to that from a reference seismometer (Guralp CMG3TD broadband seismometer located in Swindon, UK in the existing British Geological Survey monitoring network) in Fig. 5, with good agreement demonstrated. Deviations at low frequencies (<0.01 Hz) are attributed to the roll-off in the frequency response of the reference seismometer at these frequencies and the slow tilt of the platform in Bristol on which the sensor was mounted. A further example of recorded ground motion for the Oaxaca, Mexico earthquake on February 16, 2018 is shown in Figs. S8,S9.

## Summary and Future Outlook

Highly accurate and compact MEMS accelerometers are of great interest for a variety of applications. These devices can be integrated into small-diameter wireline tools and other borehole instruments for tracking fluids in the sub-surface^28^ with application to flood front monitoring^29^, carbon sequestration^30^, and mining. Surface gravity measurements are widely used for a variety of applications in exploration^31^, geotechnical surveying, identification of sub-surface voids caused by tunnelling or natural sinkholes^32^, monitoring magma flow in volcanic zones^33^, and inertial navigation. The availability of a compact, potentially low-cost and portable device significantly expands the application spectrum of gravimetry, which are limited by the expensive and fragile sensors. By reducing the cost associated with the manufacturing these monitoring instruments, arrays of tens of thousands of sensors can be deployed and wirelessly networked to address applications such as routine geotechnical surveying, monitoring fluid movement in the sub-surface (*e.g*., for management of underwater aquifers or CO_2_ storage monitoring), monitoring ground movement (slope stability) or as sensitive tilt meters for construction applications. Their response to seismic and micro-seismic events enables these devices to be deployed in large-scale monitoring networks, replacing more expensive and fragile instruments currently employed for these purposes. These results also have implications for the development of other resonant devices, such as navigation-grade MEMS gyroscopes, which require excellent bias stabilities over long integration times.

The MEMS VBA reported in this work provides several advantages over existing macroscopic instruments and consumer-grade MEMS accelerometers, including the ability to measure over a wide dynamic range ±1 g while enabling sufficient resolution for gravity measurements in the regime below 10 μGal. Furthermore, the devices are inherently robust to shock and vibration with a fundamental natural frequency of ~1 kHz, as opposed to displacement output devices based on optical or capacitive sensing that must be designed around a low operating natural frequency of <10 Hz to address measurements of similar resolution. Additionally, the resonant sensing principle scales favourably with the scaling of dimensions with, millimetre-scale devices currently in production meeting performance specifications exceeding those outlined in this first-generation device. Miniaturisation of the MEMS VBA and further integration including implementation of the associated electronics on a CMOS chip could result in portable gravimeters dissipating power <100 mW, representing a significant step change improvement in both metrics relative to current technology. The associated reduction in cost could enable a range of applications that are inaccessible for current highly sensitive, precise accelerometers.

## Methods

### Microfabrication

The fabrication process for the MEMS sensors commences with the manufacture of a VIA wafer incorporating through-silicon vias^17^ and a shallow etch to provide vertical clearance for the released MEMS devices. This wafer is then bonded^16^ to a second SOI wafer which is thinned down with the substrate layer sacrificed while leaving the device layer intact. Deep reactive ion etching^16^ is used to etch through the silicon device layer to create the device features, including the proof mass, suspension systems, vibrating beam elements and electrodes. Finally, a third silicon wafer is etched to create a cavity to house the mechanical devices and the final wafer stack (Supplementary Fig. S1) is created through wafer bonding under vacuum in an external silicon MEMS foundry.

### Electrical interface

The resonant beams are driven in closed-loop oscillation using a custom designed analogue front-end interface comprising a low-noise trans-impedance amplifier (TIA) front-end and a digital phase-locked loop (Zurich Instruments MFLI) in order to separately track the resonant frequencies of each of the two beams. The output signals are then digitized and the data is captured on a PC (National Instruments Data Acquisition platform) where a frequency counter is implemented for each channel using a DFT-based technique. A differential frequency output is calculated by subtracting the frequencies of each of the two oscillators to provide a first-order cancellation of environmental effects (see Supplementary Fig. S11).

### Environmental control and shielding

Three stages of temperature control are employed. The first stage regulates the chip temperature to a value of ±250 µ°K of a given set-point (50 °C for measurements reported in this work). The second stage controls the temperature of the surrounding analogue electronics to ±1 mK of a set-point (45 °C for measurements reported in this work) and a third stage allows for control of the temperature of entire module to a similar precision. The chip and the entire front-end electronics assembly are housed in a shielded box to prevent external electromagnetic interference.

### Earth tide data processing

The linear drift was removed from the raw data using a sliding window of duration 86400 s. A Savitzky-Golay FIR smoothing filter with polynomial order of 3 and frame length 7200 s was further applied to the linear drift corrected data. Finally, a moving mean with length 60 s was used to smooth the curve.

## Supplementary information

Supplemental manuscript.

## Data Availability

The time-series data underpinning the findings of this study are available in the University of Cambridge data repository with the identifier https://doi.org/10.17863/CAM.39454.
