# Supplementary material for: A vibrating beam MEMS accelerometer for gravity and seismic measurements
Source: Sci Rep. 2020 Jun 26;10:10415. doi: 10.1038/s41598-020-67046-x (PMC7320019; doi:10.1038/s41598-020-67046-x)
Supplement: Supplementary file 1 — Supplemental manuscript. [file 41598_2020_67046_MOESM1_ESM.pdf]

Supplementary Materials for

A vibrating beam MEMS accelerometer for gravity and seismic measurements

Authors: Arif Mustafazade[1, 2], Milind Pandit[1], Chun Zhao[1], Guillermo Sobreviela[1], Zhijun Du[2], Philipp Steinmann[2], Xudong Zou[1, 3], Roger T. Howe[4], and Ashwin A. Seshia[1, 2*]

Affiliations:

[1] Nanoscience Centre, Department of Engineering, University of Cambridge, Cambridge CB3 0FF, UK

[2] Silicon Microgravity Ltd., Cambridge Innovation Park, Waterbeach, Cambridge CB25 9GL, UK

[3] State Key Lab of Transducer Technology, Institute of Electronics, Chinese Academy of Sciences, Beijing 100190, China

[4] Department of Electrical Engineering, Stanford University, Stanford CA 94305, USA

*Correspondence to: aas41@cam.ac.uk.

Supplementary Text

Scaling analysis

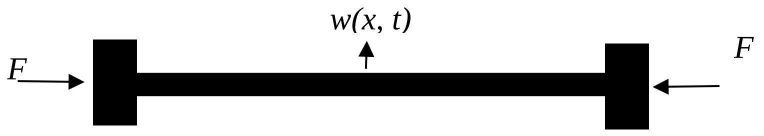

The vibrating beam can be approximated as a one-dimensional homogenous prismatic beam subject to an axial force. The transverse vibrations of the beam (with no damping) can be approximated by the following equation [S1].

$$EI\frac{\partial^4 w}{\partial x^4} - F\frac{\partial^2 w}{\partial x^2} + \rho A\frac{\partial^2 w}{\partial t^2} = 0 \qquad \text{(S1)}$$

where $w(x, t)$ is the beam displacement, E is the Young's modulus, I is the moment of inertia, F is the axial force, ρ is the density and A is the cross-sectional area of the beam. Further note, the beam displacement can be written as:

$$w(x,t) = \sum_n \phi_n(x)(a_n \cos\omega_n t + b_n \sin\omega_n t) \qquad \text{(S2)}$$

With boundary conditions approximated as: $\phi_n(0) = \phi_n(l) = \dot{\phi}_n(0) = \dot{\phi}_n(l) = 0$ (i.e. clamped-clamped) where l is the beam length and the two ends of the beam are situated at $x = 0$ and $x = l$. In the context of an accelerometer, the axial force on the beam is related to the inertial force on the proof mass under external acceleration such that $F \propto mg$. Under these assumptions, equations can be found for the mode shapes and natural frequencies of the beam under axial load [S2]. The resulting equations can be numerically solved to graph the variation of the natural frequencies as a function of external load.

A Rayleigh approximation can be applied to obtain a closed-form estimate for the natural frequency of the beam subject to axial force F [S2] which provides an excellent match to numerical results for the case of small axial loads where the contribution of the axial load to the modal

stiffness is much lower than the bending stiffness (corresponding to the applications of interest here). Nonlinear and dissipative effects are ignored (again a good assumption given that the Q factors are typically > 20000 and the beams are driven in the linear regime).

$$f_n(F) = f_n(0)\left(1+\gamma_n \frac{Fl^2}{12EI}\right)^{1/2} \text{ where } f_n(0) = \frac{1}{2\pi}\frac{\alpha_n^2}{l^2}\left(\frac{EI}{\rho A}\right)^{1/2} \quad \text{(S3)}$$

If the axial force F is replaced by the scaled inertial force ($mg*A_{\mathrm{lvr}}$) where A_{lvr} is the force amplification provided by the mechanical lever arrangement, an analytical approximation to the scale factor can be obtained as follows:

$$\frac{\Delta f}{g} = \frac{f_n(F) - f_n(0)}{g} = \frac{\gamma_n \alpha_n^2 A_{lvr} A_{mass}}{8\sqrt{3}\pi b^2}\sqrt{\left(\frac{\rho}{E}\right)} \quad \text{(S4)}$$

showing that the scale factor is related to the ratio of the area of the mass (A_{mass}) to the squared width of the vibrating beam (b), and hence is scale-invariant to first order. In practice, b can be set to the smallest dimension achievable (set by a combination of process (etch/lithography) constraints and geometric nonlinearities associated with the vibration of the beams) while A_{mass} is usually the largest dimension on the chip (set by constraints on overall chip dimensions) demonstrating compatibility of this transduction approach with miniaturization.

In the context of displacement sensing we have:

$$\frac{\Delta x}{g} = \frac{m}{k} = \frac{1}{\omega_n^2} \quad \text{(S5)}$$

where Δx is the displacement of a mass-spring gravimeter in response to gravitational input (g) along the sensitive axis. The mechanical sensitivity is clearly scale dependent (scaling as L^{-2} where L is a representative dimensional scale). This involves a fundamental trade-off between size, robustness and sensitivity *(7)*.

References:

[S1] Timoshenko, S. P., Young, D., Weaver, W., Vibration Problems in Engineering, John Wiley & Sons, 4th edition, 1974.

[S2] Albert, W. C., Force sensing using quartz crystal flexure resonators, Proc. 38th Ann. Symp. Freq. Contr., Philadelphia, PA, USA, pp. 233-239 (1984).

Supplementary Figures

Fig. S1.

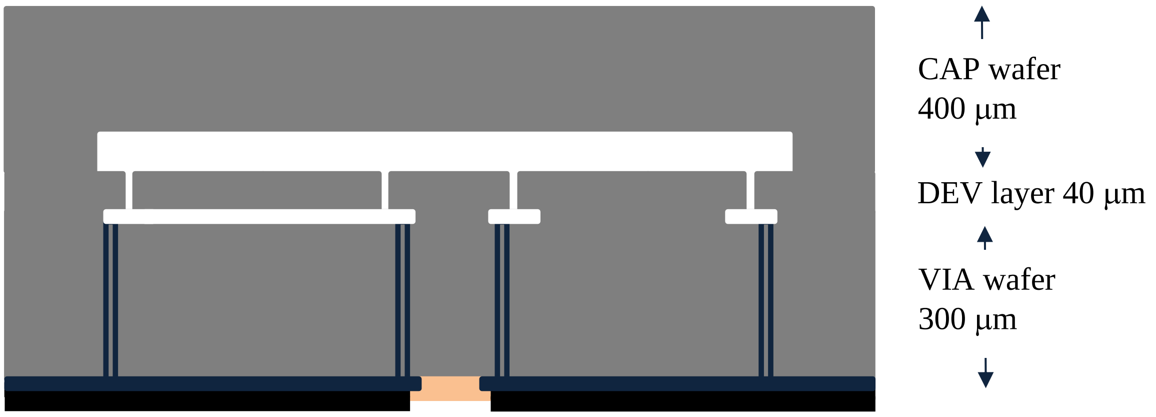

Fig. S1: Process cross-section for MEMS sensor. The devices are manufactured by bonding three silicon wafers together with the mechanical structures sandwiched between the VIA wafer (providing for electrical interconnects using high-aspect ratio trench isolation techniques) and the CAP wafer (that seals the structures in a vacuum ambient).

Process key: Single-crystal silicon, silicon dioxide, aluminium, passivation .

Fig. S2

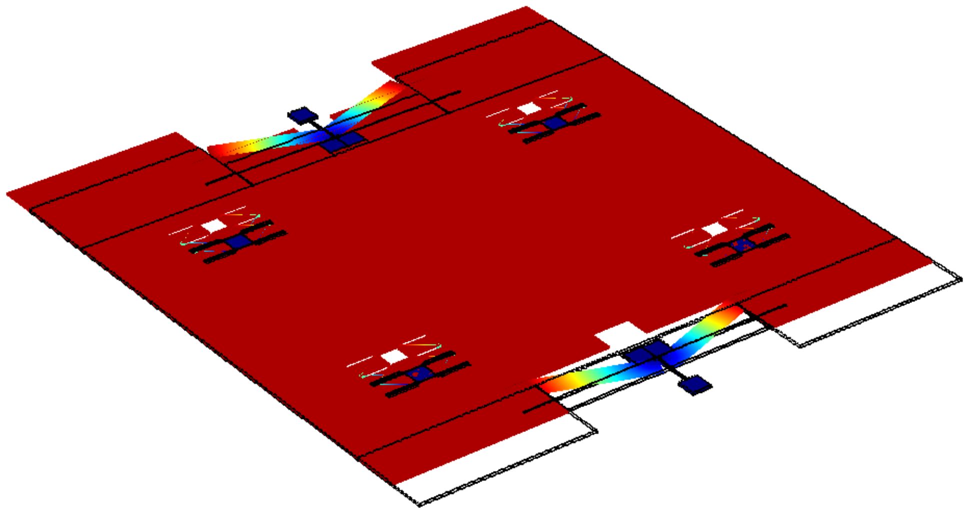

Fig. S2: Finite element model of the device illustrating the primary mechanical mode.

Fig. S3

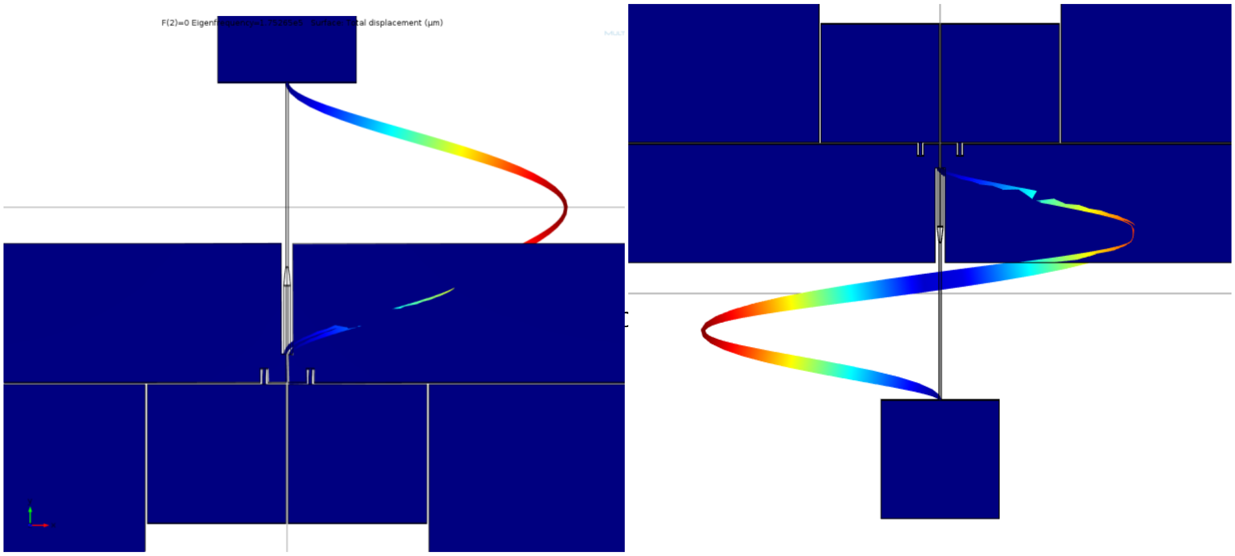

Fig. S3: Illustration of the first and second flexural modes of the vibrating beam connected to the mass (through a lever scheme) at one end and anchored to the chip substrate at the other end.

Fig. S4

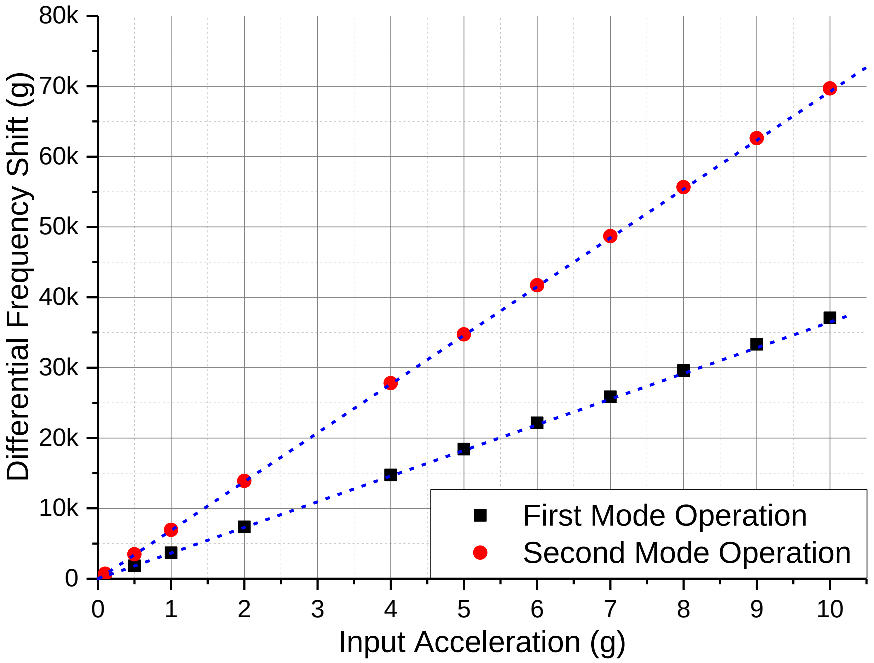

Fig. S4: Results from the finite element simulations of the differential frequency shift (the difference in the natural frequencies of the beams located on either end of the proof mass) as a function of acceleration applied along the sensitive axis.

Fig. S5

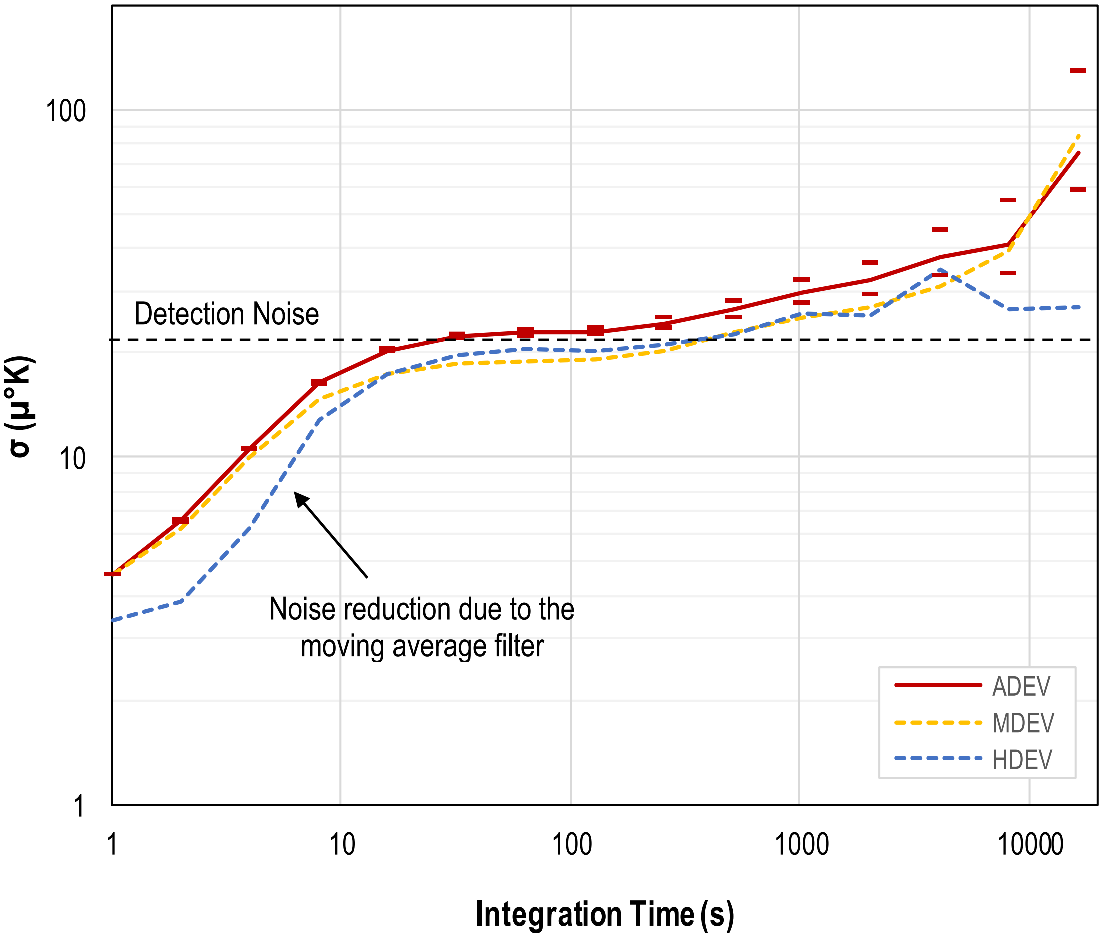

Fig. S5: Measurements demonstrating stability of the chip-level temperature (250 µ°K of a given set-point) for integration times greater than 10000s.

Fig. S6

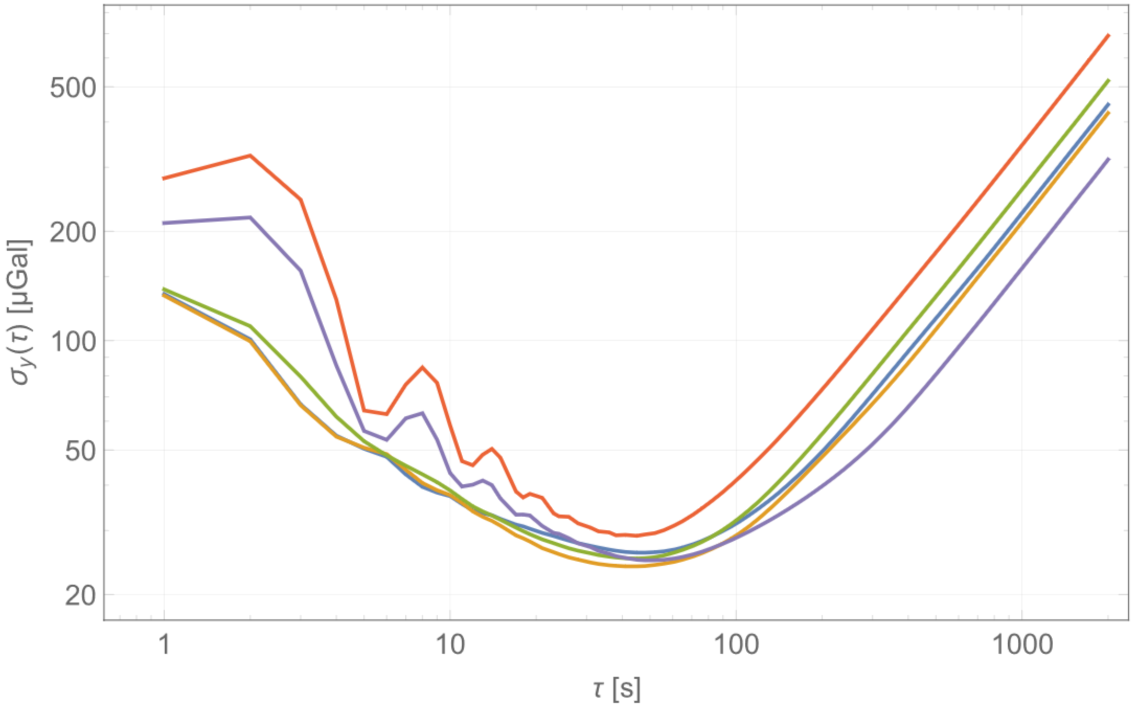

(a)

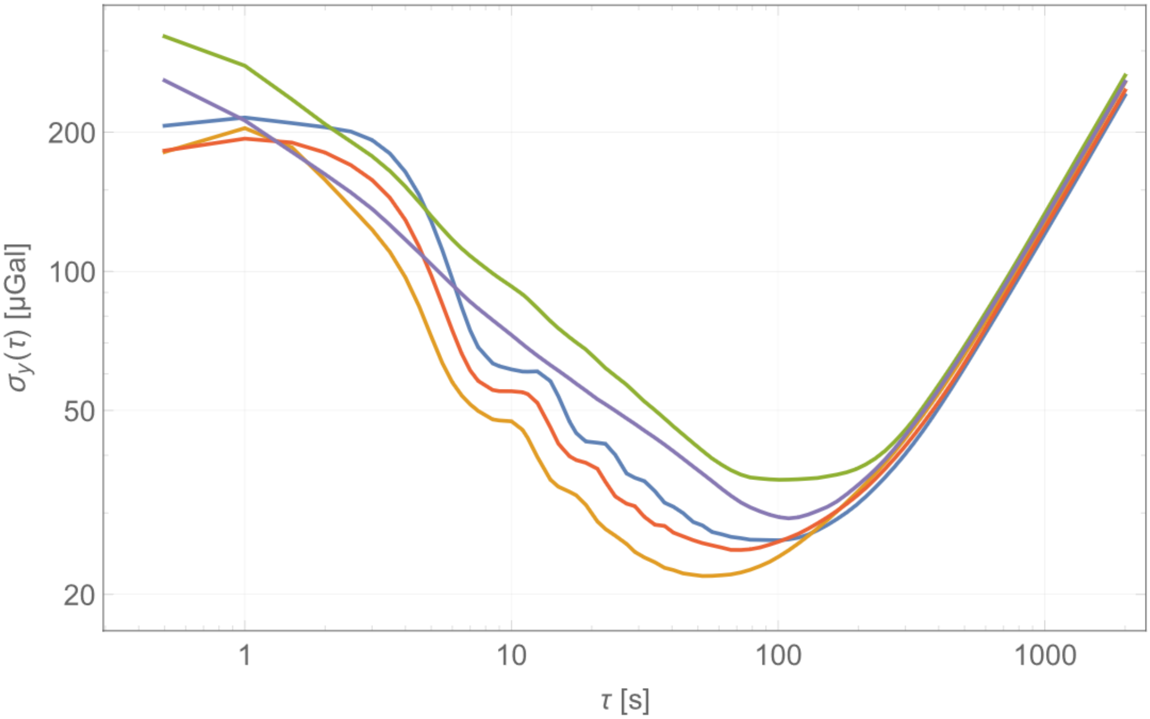

(b)

Fig. S6: Allan deviation for sensor response without drift correction recorded in (a) the low-noise facility in Eskdalemuir, Scotland, and (b) the Cambridge University lab facility corresponding to the datasets presented in Fig. 2.

Fig. S7

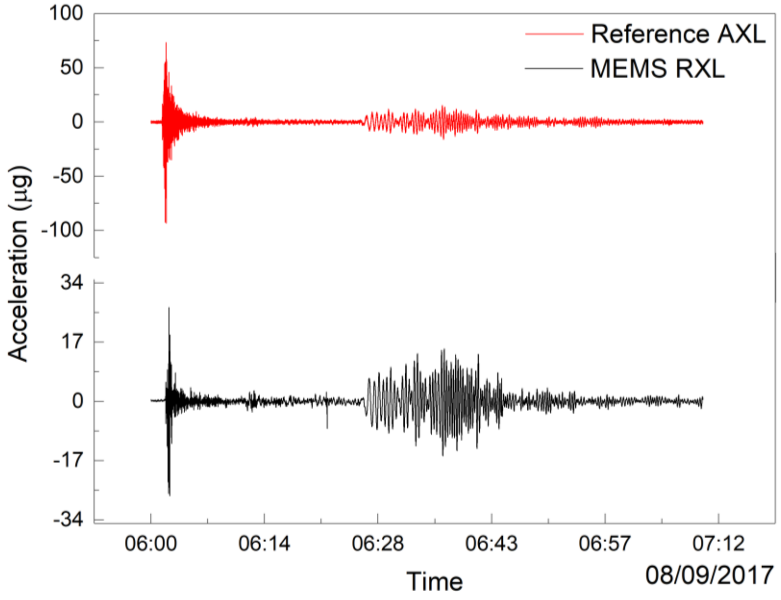

(a)

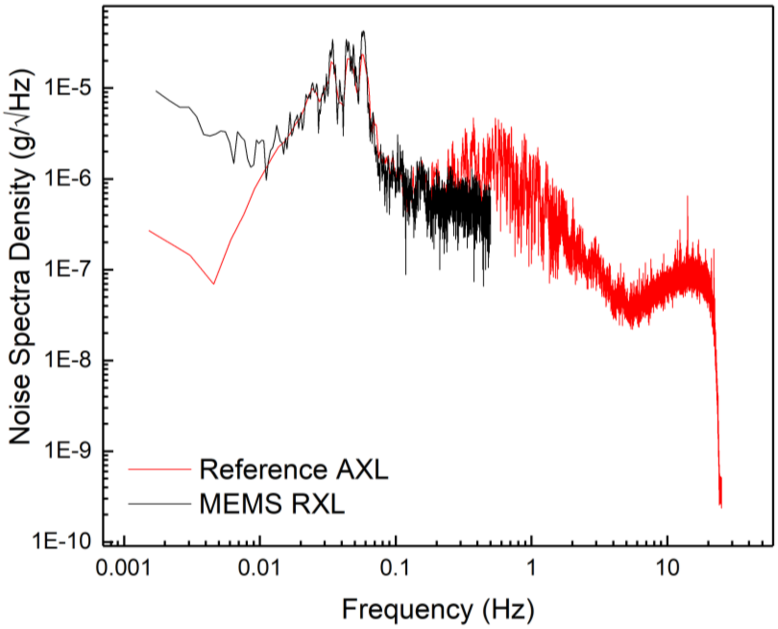

(b)

Fig. S7: Measurement of ground acceleration ((a) – time series, and (b) corresponding PSD) in a basement laboratory in Bristol, UK, and comparison of dataset to a reference seismometer located in Swindon, UK, for the 2017 Chiapas, Mexico, Earthquake (Mw 8.2) on September 8 2017. The MEMS accelerometer output was sampled at 1 Hz while the reference accelerometer was sampled at 50 Hz.

Fig. S8

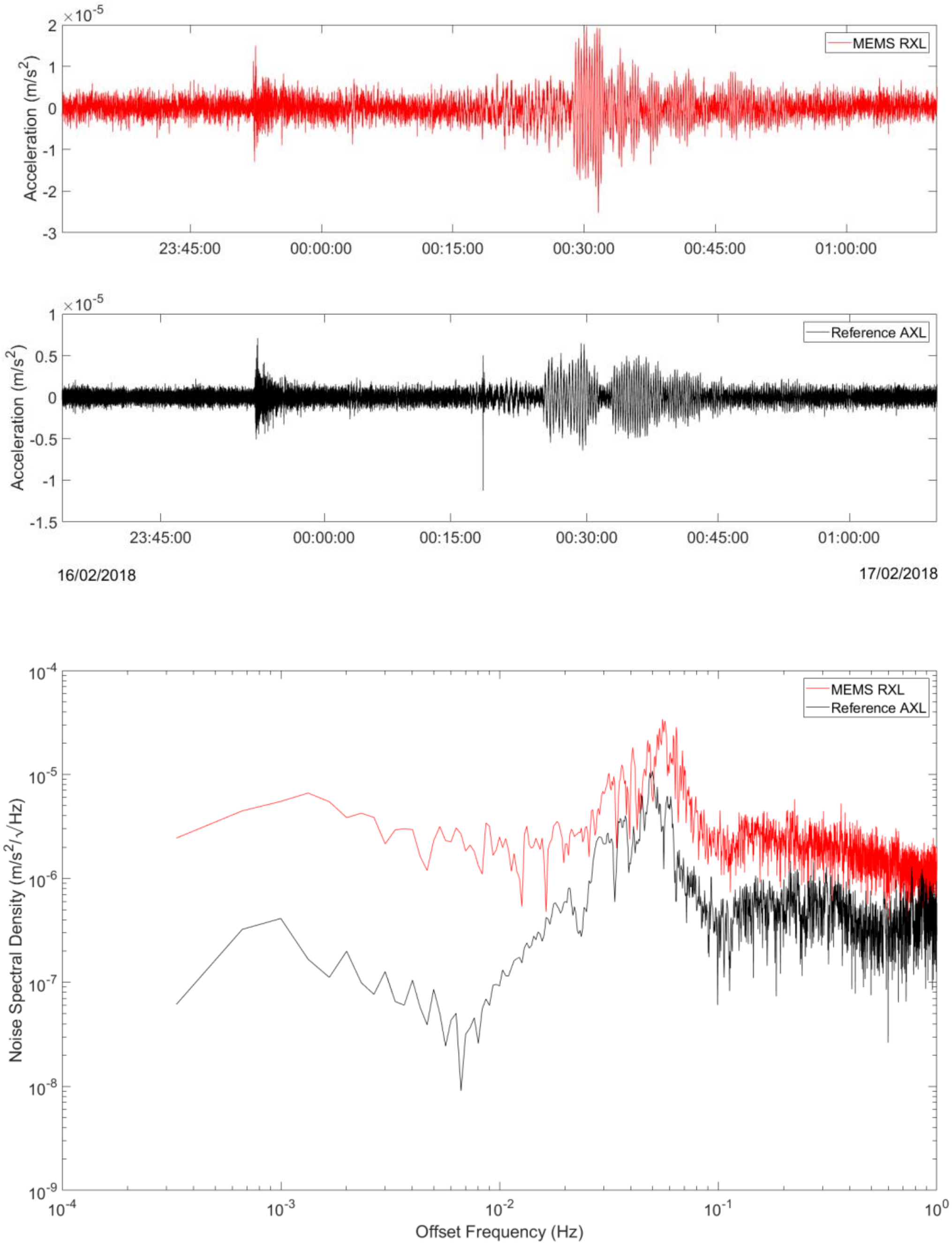

Fig. S8: Comparison of the time-series and noise-spectral density of the MEMS seismometer based in Cambridge, UK relative to a reference broadband seismometer (Guralp CMG3TD) integrated as part of the BGS network in Elham, UK in response to the 16th February 2018 earthquake in Oaxaca state in southern Mexico (Mw 7.2). Note that the broadband seismometer is configured to operate over a bandwidth between ~0.01 Hz – 50 Hz.

Fig. S9

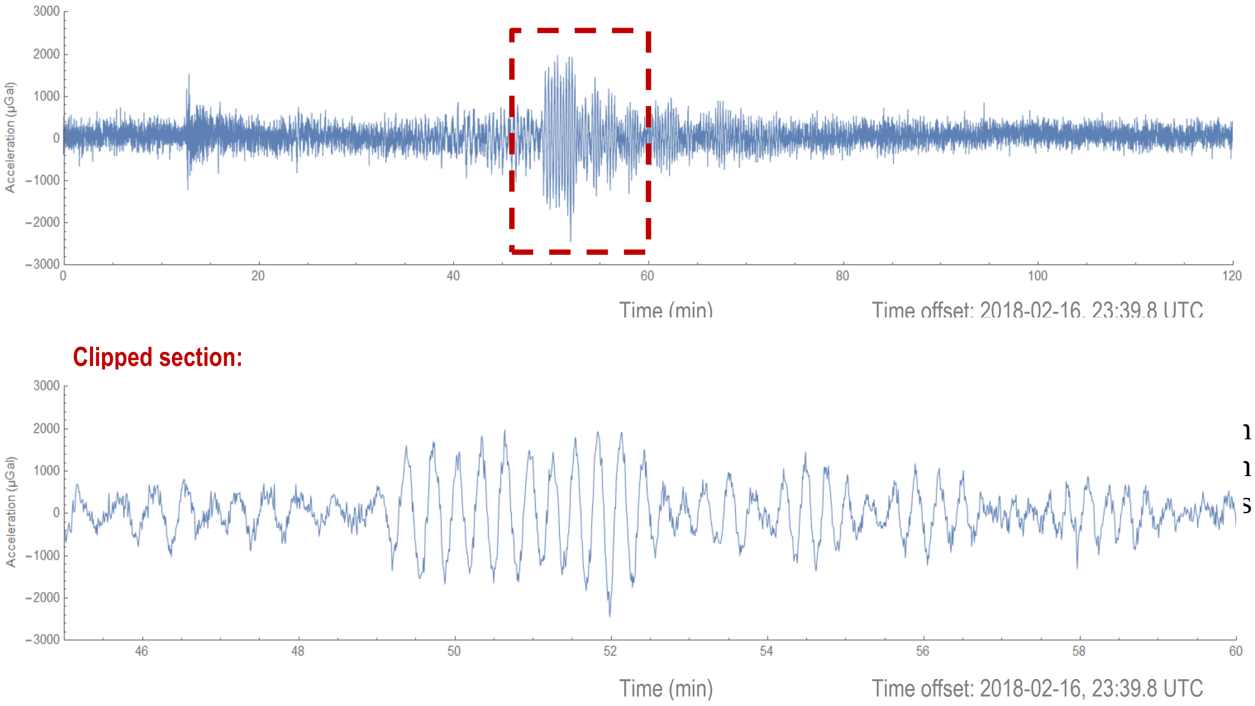

Fig. S9: Record of ground oscillations as measured by the MEMS instrument (situated in Cambridge, UK) in response to the 16th February 2018 earthquake in Oaxaca state in southern Mexico (Mw 7.2). A clipped-section of the time-series corresponding to the long-period oscillations is shown in the lower panel.

Fig. S10.

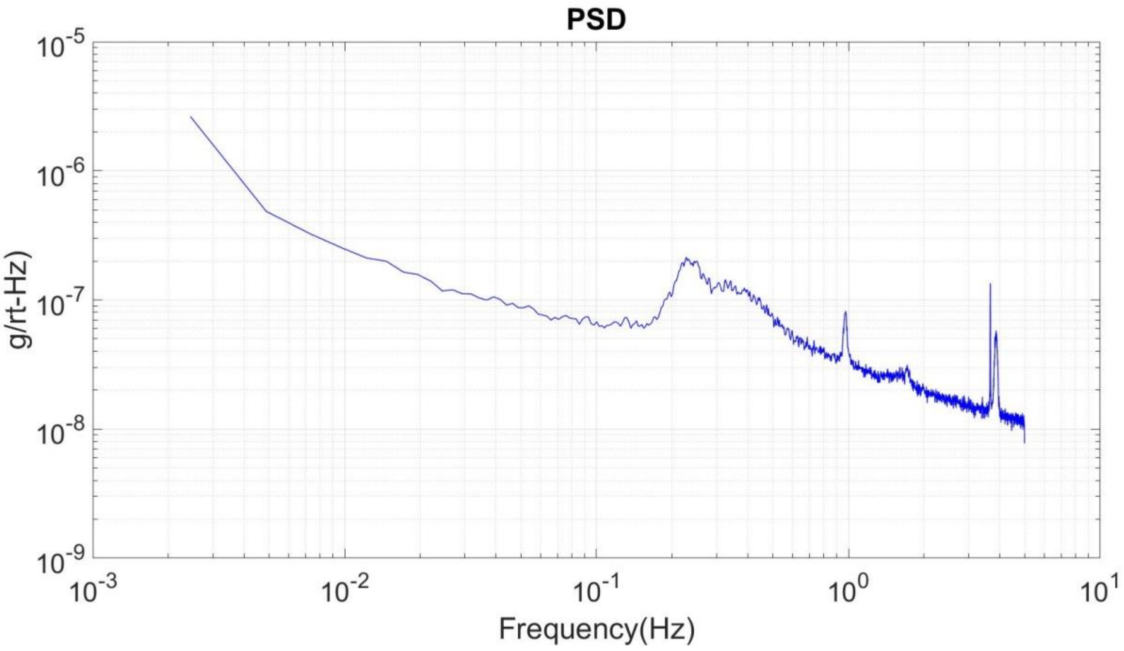

Fig. S10: Noise power spectral density for the MEMS sensor with the sensitive axis oriented in the horizontal configuration while integrating two levels of temperature control. The sampling is conducted as 10 samples/s and the noise floor is 25 nano-g/rt-Hz at 1.5 Hz.

Fig. S11.

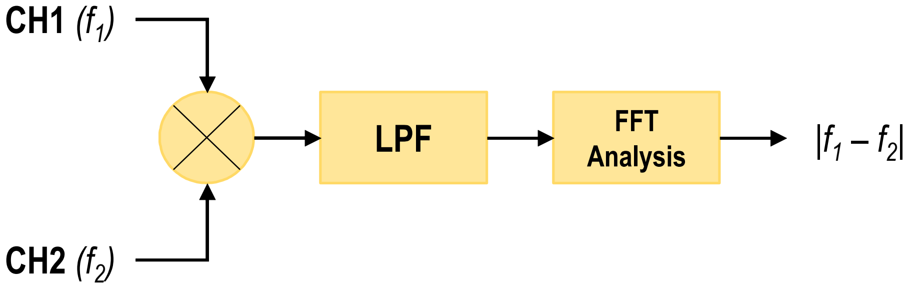

Fig. S11: Schematic implementation of the frequency counting approach for the measurement of acceleration.
